# Deterministic Versus Stochastic Cell Polarisation Through Wave-Pinning

**DOI:** 10.1007/s11538-012-9766-5

**Published:** 2012-09-07

**Authors:** Georg R. Walther, Athanasius F. M. Marée, Leah Edelstein-Keshet, Verônica A. Grieneisen

**Affiliations:** 1 Computational & Systems Biology, John Innes Centre, Norwich Research Park, Norwich, NR4 7UH UK; 2 Mathematics Department, The University of British Columbia, Vancouver, BC V6T 1Z2 Canada

**Keywords:** Rho GTPase, Polarization, Wave-pinning, Stochastic model, Local perturbation analysis

## Abstract

Cell polarization is an important part of the response of eukaryotic cells to stimuli, and forms a primary step in cell motility, differentiation, and many cellular functions. Among the important biochemical players implicated in the onset of intracellular asymmetries that constitute the early phases of polarization are the Rho GTPases, such as Cdc42, Rac, and Rho, which present high active concentration levels in a spatially localized manner. Rho GTPases exhibit positive feedback-driven interconversion between distinct active and inactive forms, the former residing on the cell membrane, and the latter predominantly in the cytosol. A deterministic model of the dynamics of a single Rho GTPase described earlier by Mori et al. exhibits sustained polarization by a wave-pinning mechanism. It remained, however, unclear how such polarization behaves at typically low cellular concentrations, as stochasticity could significantly affect the dynamics. We therefore study the low copy number dynamics of this model, using a stochastic kinetics framework based on the Gillespie algorithm, and propose statistical and analytic techniques which help us analyse the equilibrium behaviour of our stochastic system. We use local perturbation analysis to predict parameter regimes for initiation of polarity and wave-pinning in our deterministic system, and compare these predictions with deterministic and stochastic spatial simulations. Comparing the behaviour of the stochastic with the deterministic system, we determine the threshold number of molecules required for robust polarization in a given effective reaction volume. We show that when the molecule number is lowered wave-pinning behaviour is lost due to an increasingly large transition zone as well as increasing fluctuations in the pinning position, due to which a broadness can be reached that is unsustainable, causing the collapse of the wave, while the variations in the high and low equilibrium levels are much less affected.

## Introduction

Many eukaryotic cell types undergo directed movement in a variety of scenarios. Such motility is important in embryogenesis (Charest and Firtel 2007), wound healing, immune surveillance (Ridley et al. 2003), and cancer metastasis (Ridley et al. 2003). As a first step in this process, cells polarise, forming a distinct front and rear distinguished by biochemical profiles of signalling molecules that regulate lamellipodial extension (Ridley 2006). An important part of that internal polarizing biochemistry is based on the activity and distribution of Rho GTPases. These switch-like signalling proteins exhibit a distinct active (GTP, membrane-bound) form and an inactive (GDP) form that is largely cytosolic. Only the active, GTP-form is able to interact with downstream effectors to exert its biological function. Interchange between these two forms is mediated by GTPase-activating proteins (GAPs), which augment inactivation, and guanine nucleotide exchange factors (GEFs), which facilitate activation. It has been established that the active form increases its own rate of activation via various self-recruitment mechanisms (Raftopoulou and Hall 2004; Li et al. 2003). While the active form binds the plasma membrane, the inactive form can be both in the membrane or released to the cytoplasm, a process which is positively regulated by binding to guanine nucleotide dissociation inhibitors (GDIs).

When a cell is stimulated, some Rho GTPase activity (notably, Cdc42 and Rac1) is focused at the leading edge (Ridley et al. 2003), inducing localized actin polymerization that generates protrusive forces propelling the cell (Raftopoulou and Hall 2004). Here, we are concerned about the onset of polarity and its maintenance, thus focusing only on the polarization of the Rho pattern, and not on the downstream remodelling of the cytoskeleton (or possible feedbacks that this might generate).

**Fig. 1:**
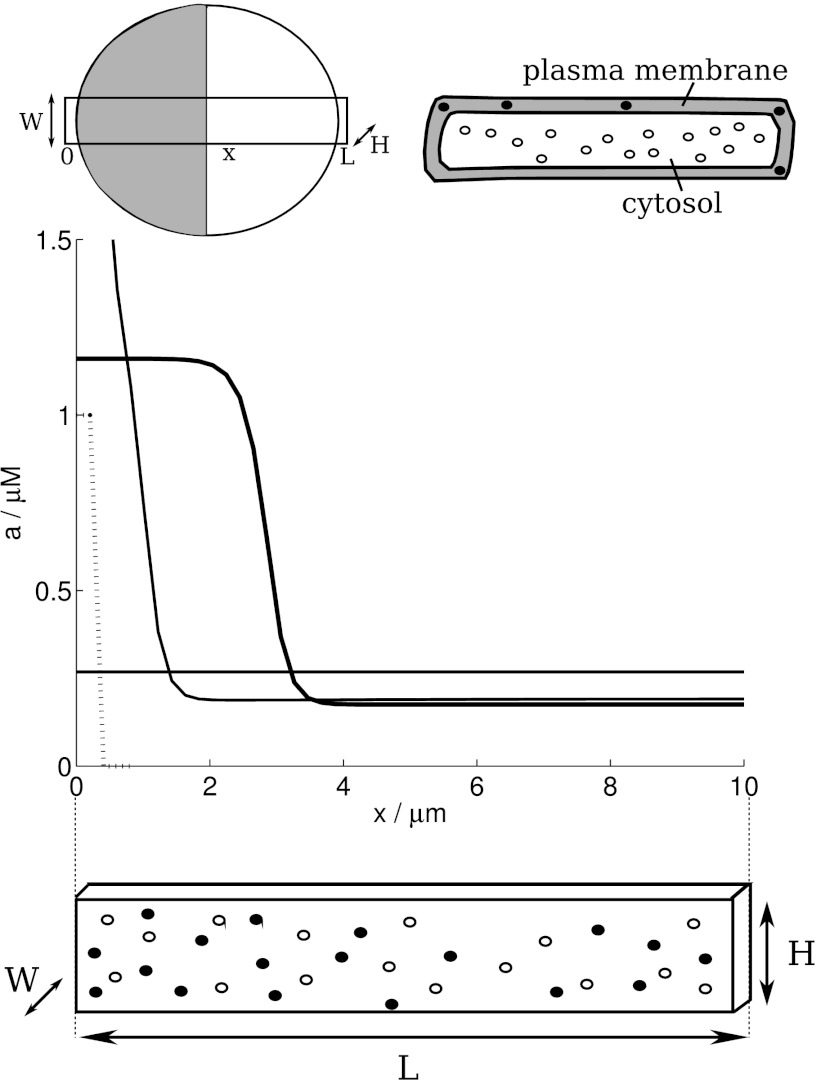
*The modelled cell*. (*Top*): Schematic diagrams of a cell showing the “slab” of length *L*, height *H*, and width *W* in a top–down view and two side-views. The model distinguishes membranous (*A*, *solid circles*) and cytoplasmic (*B*, *open circles*) proteins only by their distinct rates of diffusion. A typical “polarisation” state is shown in *grey/white* in the top–down view. (*Bottom*): In the deterministic polarisation model (1a), (1b) proposed by Mori et al. (2008), a small stimulus (*dashed line*, not to scale) produces a pinned wave (*solid black line*). The notations *x*/μm and *a*/μM along the graph axes indicate that the *x*- and *y*-axis variable carry units of μm and μM, respectively. Same notations have been used throughout the paper

Based on general interactions between Cdc42, Rac, and Rho, and taking into account known parameters for the kinetics and diffusion of the active and inactive forms, we have shown earlier that sustained polarization within a cell is possible, even when the well-mixed system has only one equilibrium, and in the spatial setting this equilibrium is stable against both homogeneous and small non-homogeneous perturbations (Marée et al. 2006; Jilkine et al. 2007). Later, we determined the mathematical essence of the mechanism by studying a reduced deterministic model of cell polarization, coining it “wave-pinning” (Mori et al. 2008). It remains, however, unclear to what extent stochasticity at low molecule numbers can influence the potential of the mechanism to initiate and sustain polarity within the cell. We therefore compare and contrast the deterministic and the stochastic version of the core model for wave-pinning. A simple 1D geometry in which this generic Rho GTPase can be studied is shown in Fig. 1, where the organelles and nucleus are omitted, *L* is a cell diameter, and the chemical system is modelled by a two-component reaction with distinct rates of diffusion *D*_*a*_≪*D*_*b*_ across *L*, since proteins diffuse much more slowly in the lipid membrane than in the cytosol. Here, *A* is the active and *B* the inactive small GTPase (with concentrations *a*(*x*,*t*) and *b*(*x*,*t*)). The height *H* and width *W* of the compartment are assumed to be reasonably small, so gradients are described in the *x* direction for *x*∈[0,*L*], *t*≥0. The system of reaction-diffusion equations of the deterministic GTPase model in Mori et al. (2008) is 
1a
1b Here, *f*(*a*,*b*) is the rate of GTPase interconversion. Equations (1a), (1b) are taken with no-flux boundary conditions: *a*_*x*_(0)=*a*_*x*_(*L*)=0, *b*_*x*_(0)=*b*_*x*_(*L*)=0 and the inter-conversion rate is modelled to include auto-activation of *A* (through a positive feedback of *A* onto its own production):

2 $$ f(a,b)=k_{0}b+\frac{\gamma a^{2}}{K^{2}+a^{2}}b-\delta a, $$

where *k*_0_ and *δ* denote the basal rates of activation and inactivation of *A*, respectively, *γ* is the rate of maximal feedback strength, and *K* is the concentration of *A* leading to a half-maximal feedback level.

We first briefly describe the deterministic aspects of this model, and build on the previous analysis by introducing a local perturbation analysis that leads to insights on how the initiation of polarisation depends on the parameters and on the total amount of molecules. We then explore how the polarisation mechanism reacts when only a limited number of molecules is available and stochasticity starts to impact on the polarisation state of the cell. To do so, we describe and analyse an analogous stochastic version (low copy number regime) of the same model. We confirm our stochastic implementation by showing that simulating large molecule and lattice numbers approaches the thermodynamic limit. To analyse the equilibrium behaviour of the stochastic system, we introduce statistical tools which provide us with intriguing insights regarding the dynamics in the low copy number regime, namely it being dominated by spatial fluctuations of the transition zone rather than temporal fluctuations in the activity level, and loss of polarity due to the region of high activity, through stochastic fluctuations, reaching a broadness that is unsustainable, causing the sudden collapse of the whole wave. Bifurcation analysis of a simplified model of a pinned wave provides us with a straightforward rationale for the behaviour of the stochastic system close to the point where the wave is lost due to stochastic fluctuations.

## Deterministic Behaviour

### Wave-Pinning

Given appropriate conditions, within model (1a), (1b) a stimulus-pulse of GTPase located within an otherwise homogeneous domain, for example at one end of the cell, leads to the formation of a travelling wave of activation that slows down and stalls, delimiting a spatial region of activation, i.e. creating a robustly polarised cell (Mori et al. 2008). In our simulations, this initial pulse is captured by a first-order reaction converting *b* to *a* within a small domain of the cell. This is done through the term *k*_*s*_ *b*(*x*,*t*), which is both added to Eq. (1a) and subtracted from Eq. (1b). The wave-pinning regime depends on the relative rates of diffusion and the total amount $T = \int_{0}^{L}a(x,t)+b(x,t)\,dx$ in the system. The mechanism of wave-pinning can be attributed to the following: relatively rapid diffusion of *b* (*D*_*b*_≫*D*_*a*_) leads to a more-or-less constant level of *b* over the cell, while the existence of three roots of *f*(*a*,*b*) for the fixed well-mixed equilibrium *b* level allows for a sufficiently large local perturbation in *a* to locally reach a distinct activity level (a process that we have coined “*Δ*-perturbability”, see below). Mass conservation ensures that, while this peak of increased *a* levels expands its domain over the cell with its front propagating like a wave, the more-or-less homogeneous level of *b* drops. This global decrease of *b* slows down and eventually limits the spatial propagation of the wave, pinning it at an equilibrium position (Mori et al. 2008). Even though wave-pinning requires the existence of three roots of *f*(*a*,*b*) for fixed *b* level, it is important to realize that it is not a consequence of bi-stability and subsequent front propagation between two stable states. (Note that in reaction-diffusion systems, the terminology bi-stability is used to denote cases in which the corresponding well-mixed system has two distinct stable steady states.) The well-mixed ODE system has only one equilibrium, and in the PDE this equilibrium is stable against both homogeneous and small non-homogeneous perturbations. Nevertheless, in the spatial setting a sufficiently large local perturbation can trigger the travelling wave, which subsequently stalls, giving rise to sustained polarity.

### Wave-Pinning Versus Propagation Failure

Because we compartmentalize space in this study to perform stochastic simulations, it is relevant to introduce yet another mechanism, coined propagation failure (Britton 1985; Keener 1987). As a possible source of confusion, propagation failure has previously also been referred to as “pinning of waves” (Fáth 1998), thus evoking the need to emphasize its clear distinction from “wave-pinning” as defined in Mori et al. (2008).

Propagation failure describes a specific phenomenon that can be observed in bistable systems in which travelling waves fail to propagate when space is discrete. This may occur when both the wave velocity is low and the discretisation of the space is coarse (relative to the diffusion coefficient) (Keener 1987; Fáth 1998). Under such conditions, propagation failure can manifest itself if, at the location of the wave front, the diffusive flux from one sub-domain into the next becomes insufficient to bring the levels of that sub-domain above the threshold required for the amplification and subsequent propagation of the wave. In contrast, the phenomenon of wave-pinning does not require a discretised space. Instead, when the triggered wave spreads over the domain, the velocity of the wave decreases, because of the drop in the available inactive form that is used up by being converted into the active form. Nevertheless, we here find that both phenomena become coupled to one another when space is discretised. Due to the slowing down of the wave during the wave-pinning process, inevitably the velocity of the wave eventually becomes sufficiently low that propagation failure will occur within coarse grids. Consequently, when we discretise space in this study, which we do in both numerical PDE simulations and in Gillespie simulations, propagation failure occurs for large sub-domain sizes as well as low diffusion rates.

Given that the sub-division into compartments is a computational method, but does not represent a biological property of the cell, we will ensure below that propagation failure does not play a role in the dynamics presented in this paper nor influences the biological insights we derive here. This brings us to the next issue, which is how to distinguish propagation failure from wave-pinning, given that in both cases the wave stalls.

### Analysis of Polarity Initiation

The full bifurcation analysis of any system of partial differential equations (PDEs) is a challenging undertaking. While Mori et al. (2011) focused on the requirements of the travelling wave to stop, we will here discuss an analysis regarding the potential to initiate polarity and a travelling wave, in which we probe the homogeneous state of the cell with a local perturbation. In short, we ask what happens if a local perturbation is introduced to a resting cell (being at a uniform steady state), by observing whether such a perturbation will diverge to a distinct local equilibrium (eventually causing polarisation through wave-pinning), or alternatively dampen out, returning to the rest state corresponding to the global state of the cell. This analysis provides a straightforward test whether a (sufficiently large) perturbation can “invade” the initially uniform steady state solution. We refer to this reduced model as the “local perturbation analysis” (LPA) model, or system, as it allows us to study invasion criteria for a local perturbation of any given amplitude. (Note that such *Δ*-perturbability does not directly imply sustained polarisation through wave-pinning, see below.)

To address the onset of polarisation without having to deal with the full complexity of the PDEs, we break down the spatial system into two effective compartments, one corresponding to the levels of the active and inactive form at the site of the local perturbation, and another corresponding to the global values over the rest of the cell. The simplified representation of the deterministic system is given in Eqs. (4a), (4b), used to predict the total amount of small GTPase *T* for which to expect initiation of polarisation and wave-pinning. This analysis allows us to compare results from the deterministic and the stochastic version of the model later on. For the local perturbation analysis, we make the following assumptions and approximations to the PDE model:

- We ask whether the value of *A* at a site of the localized pulse *a*_*L*_(0) will diverge from the uniform global concentration of active GTPase *a*_*G*_(*t*). Since this active form has a very low rate of diffusion, we consider the limit *D*_*a*_≈0 and treat *a*_*L*_(*t*) as a purely local variable, that can vary independently from *a*_*G*_(*t*). This is equivalent to assuming that any perturbation in *A* will be spatially confined to the site of the perturbation and will initially evolve independently of the rest of the domain.
- Since the inactive GTPase *B* has a relatively fast rate of diffusion, we take the limit at which *D*_*b*_≈∞ and consider *b*(*t*) to be a purely global variable (*b*_*L*_(0)=*b*_*G*_(*t*)≡*b*(*t*)). Restated, any local perturbation in *B* caused by the local perturbation in *A* will be instantly adjusted to the global, homogeneous concentration profile. This leads to the following LPA model:

3 $$ \frac{da_{L}}{dt}=f(a_{L},b), \qquad\frac{da_{G}}{dt}=f(a_{G},b), \qquad\frac{db}{dt}=-f(a_{G},b). $$

Furthermore, given that we consider only a narrow initial pulse of activation that hardly affects the overall cell levels, it is reasonable to approximate $T(t)\approx\int_{0}^{L}a_{G}(t)+b(t)\,dx\approx \mbox{constant}$, so *b*(*t*)≈(*T*/*L*)−*a*_*G*_(*t*). Eliminating *b*(*t*) by conservation leads to a system of two ODEs: 
4a
4b


**Fig. 2:**
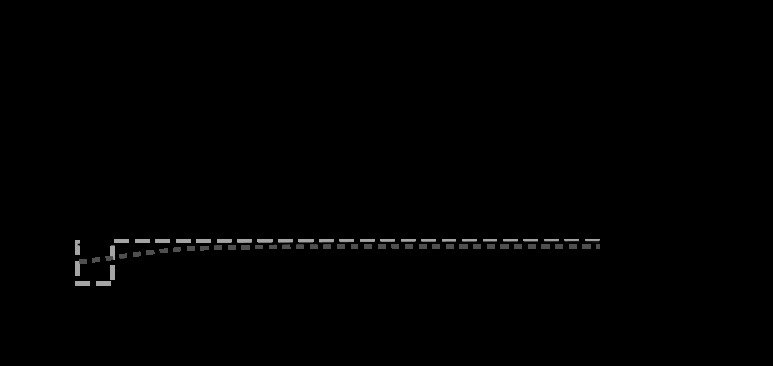
*Local perturbation analysis*. Schematic representation of the deterministic local pulse analysis (LPA). In our reduction, we introduce a local perturbation *Δa*, and consider the evolution of the local variable *a*_*L*_, the inactive protein (represented by the *dotted lines*, evolves to the *straight black line*, such that *b*_*L*_=*b*_*G*_=*b*), and the active protein in the rest of the cell, *a*_*G*_

The approximations required for the polarity-invasion analysis are depicted in Fig. 2. Note that since the activating pulse is confined to a sufficiently small sub-section of the domain its variation will not affect the total amount of inactive form and, therefore, the dynamics of *B* solely depends on the global level of *A* in the extended domain. We can use such approximations to address the following questions: under what circumstances would a localized pulse of activation grow in magnitude compared to the surrounding levels? How large should the amplitude of the stimulus be to trigger a new state (e.g. depicting an initial polarisation)? And, if a new, bounded state exists, what values do we expect it to have? These answers depend on the parameters and on the total number of molecules (i.e. amount of small GTPase) in the system. Note that we do not restrict attention to small amplitude perturbations (as is common in linear stability analysis, e.g. of reaction-diffusion equations).

**Fig. 3:**
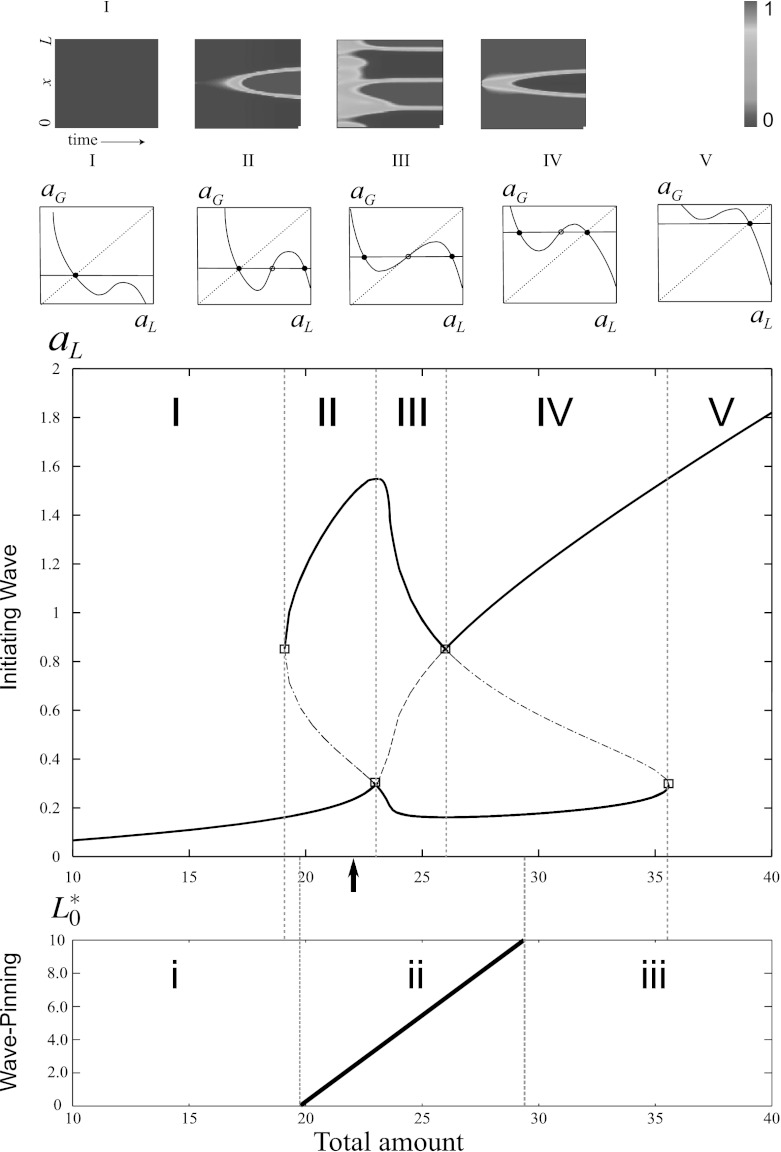
*Regions of polarisation behaviour in the deterministic model*. *Middle graph*: Bifurcation diagram of the reduced deterministic model system, Eqs. (4a), (4b), showing steady-state local activated form, *a*_*L*_, versus the total amount of material in the domain (*T*). Five distinct regimes of behaviour are found, as explained in the text. *Top row*: *colour* plots of the solutions of the PDEs, starting close to the uniform steady state in the given region. In these space-time plots, time axis is horizontal and space axis is vertical. Activity level is depicted using a *colour* gradient, with *red* indicating the highest and *blue* the lowest activity levels. *Middle row*: phase plane behaviour in the *a*_*G*_ *a*_*L*_ planes, showing the number and stability of steady states of the reduced system in each of the regions. In regions I and V, only a uniform level of global activity is stable, and no pulse or stimulus can grow. In the intermediate regions, only a sufficiently large pulse (II), or a sufficiently low dip (IV) can grow. In region III, the homogeneous state is unstable to any perturbation, and a variety of patterns can form, depending on initial conditions. *Bottom graph*: Wave-pinning position as a function of *T*. In regions *i* and *iii* no sustained polarised state is possible, while in region *ii* wave-pinning occurs, with the position of pinning, *L*_0_^∗^ monotonically increasing with *T* (Color figure online)

The LPA system (4a), (4b) can be studied in several ways, e.g. via the *a*_*G*_ *a*_*L*_ phase plane and ODE bifurcation analysis. Performing such analysis reveals five qualitatively distinct regions of polarity behaviour, depicted in Fig. 3. The behaviour in each region can be captured with a sequence of corresponding *a*_*G*_ *a*_*L*_-phase plane diagrams. On the bifurcation diagram, we plot the steady state value *a*_*L*_ versus the bifurcation parameter *T*, representing the total amount of the small G-protein. The five distinct regimes of behaviour (labelled I–V) are separated by two saddle-node and two transcritical bifurcations. The shape of the curve traversing the diagram from lower left to upper right is shared with the bifurcation curve that would be obtained when the PDE system is taken to be well mixed (*D*_*a*_=*D*_*b*_=∞). It represents a steady state where *a*_*G*_=*a*_*L*_ (unpolarised cell, “rest state”). The two actually coincide, meaning that the equilibrium level in the local patch is neither lower nor higher than the uniform global background level of *a*_*G*_. Note that for any equilibrium found in the well-mixed case, there should exist a corresponding equilibrium *a*_*G*_=*a*_*L*_ in the LPA model. The stability of the equilibrium, however, can change (from stable to unstable). For example, while the equilibrium in the well-mixed case is always stable, the portion of that curve in Region III of the LPA model is unstable. We indicate the line *a*_*G*_=*a*_*L*_ in the *a*_*G*_ *a*_*L*_ planes, using a dashed grey line, representing the absence of any local perturbation.

We now explain how to interpret the diagram and its implications for polarity behaviour. (I) In region I the total amount of molecules is low (*T*<19.09) and a regime is found in which only one steady state value *a*_*L*_=*a*_*G*_<0.2 exists. That state of low activation is unresponsive to stimuli, and no pulse can “invade”. The time-space plot of *a*(*x*,*t*) stays at, or rapidly returns to, a low uniform level (solid curve) no matter how large the amplitude of an applied stimulating pulse. That is, a cell will not polarise when stimulated, it remains unpolarised, in a rest state. (II) This is the region corresponding to the deterministic regime of cell polarisation (wave-pinning). Here, for an intermediate level of substance 19.09<*T*<23.0, there are three co-existing equilibria of Eqs. (4a), (4b), the outer two of which are stable. A value of *a*_*G*_ corresponding to the lower branch can be “invaded” by a local pulse, provided its amplitude is large enough to surpass a threshold depicted by dashed curve in region II. The lower branch corresponds to the *a*_*G*_=*a*_*L*_ equilibrium, i.e. to a cell that is in a homogeneous rest state. Thus, the rest state is stable against small perturbations, but sufficiently large perturbations can polarise the cell. Note that as the total amount increases, the required amplitude to trigger polarisation decreases sharply, so that close to but just short of *T*=23.0 a pulse of very small size can lead to polarisation. We see that the full PDE solutions (top panels in Fig. 3) show the invasion of such a pulse in this regime, which becomes established at a finite amplitude over some fraction of the domain. (III) Other patterned states (e.g. with one or more patches of active GTPase) occur in this region. For 23.0<*T*<25.99, the global steady state *a*_*G*_=*a*_*L*_ is un-stable to any perturbation. Here, small amplitude noise or a pulse of small magnitude will disrupt the global state leading to other patterned states. This kind of behaviour is typical of a Turing instability. Indeed, the full PDE solution (with random noise initial conditions such that the total amount falls in this range) produces patterns with multiple peaks. (IV) For even higher values, 25.99<*T*<35.58, the total amount of GTPase is so high that the global level of activation is at an elevated steady state level (highest solid branch of the diagram). Here, an invading “pulse” has to locally deactivate a region in order to “invade” (i.e. the pulse is a dip below the uniform global level). The amplitude of that “dip” must cross the threshold (dashed portion of curve) to trigger the polarisation, as otherwise it decays back to the uniform activation level. As shown in the solution of the PDEs, a dip of sufficiently large amplitude leads to a stable local patch of depressed activity in an otherwise high global level of activity. (V) Finally, above *T*>35.58 the potential of polarisation is lost again. That is, no pulse or dip can invade, and the uniform global state is one of high activity everywhere in the domain.

The LPA does not address the question at which position the wave will be pinned, but rather if a wave can be triggered and how high it will become. The next question therefore is at which position along the cell length the wave stalls. We indicate the wave position by *L*_0_, and the equilibrium value of *L*_0_ at which the wave stalls *L*_0_^∗^. In Mori et al. (2008), the wave-pinning position has been derived mathematically for the limiting case of an infinite difference in diffusion rate between the active and inactive form (i.e. using a sharp front approximation). In the bottom panel of Fig. 3, we show the steady state value *L*_0_^∗^ as a function of *T*. Regarding the wave-pinning itself, three regions of qualitatively different behaviour can be discriminated. In regions *i* and *iii*, no stable polarity can be found, because any wave would completely retract or expand over the whole domain, respectively. In contrast, in region *ii* we find the possibility of a stable co-existence of a high and a low state is found. Note that the pinning position *L*_0_^∗^ depends on the value of *T*. Importantly, the figure shows that the interval of sustained polarity is smaller than the interval of regions II–IV. It illustrates that even when a wave can be triggered, it does not always follow that it can also be sustained.

Note that both the results of the LPA and of the wave-pinning position act as an approximation to the actual PDE behaviour, where actual rates of diffusion are finite and initial conditions can affect whether initially a single peak or several peaks emerge. However, it correctly captures the basic boundaries that determine its potential to polarise, the minimum perturbation amplitude required to do so, the expected values to be reached at the local perturbation, and the position at which the wave pins. We will show how this brings valuable insights when interpreting the role of stochasticity in cell polarisation by wave-pinning.

## Stochastic Version

Next, we ask how the same polarisation mechanism would behave in the low copy number regime. We ask under what conditions a stochastic equivalent of the deterministic model still presents wave-pinning, i.e. after triggering the formation of sustained regions within the cell with respectively low and high levels of the active form, and if our approach predicts biologically relevant conditions under which wave-pinning may be unsustainable in live, noisy cells.

**Fig. 4:**
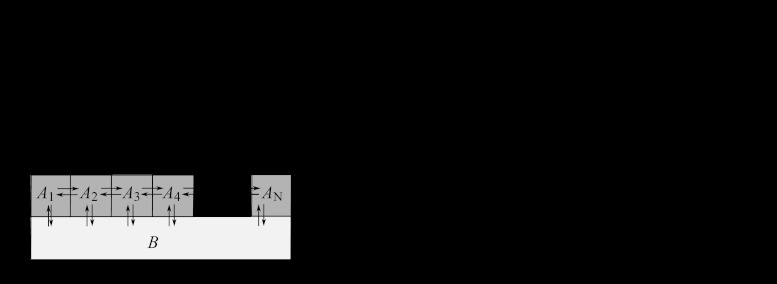
*Spatial set-up of the stochastic simulations*. The membrane is divided into *N* equally wide well-mixed sub-domains. Diffusion within the membrane is modelled as first-order reactions while we assume that *B* diffuses fast enough to warrant modelling it as occupying a well-mixed cytoplasmic pool. Dimensions *H* and *W* associated with the domain of length *L* are necessary for conversion of concentrations to numbers of molecules but do not play any other role: all stochastic simulations are one-dimensional

For stochastic simulations of our system, we resort to the stochastic formulation of chemical kinetics (McQuarrie 1967), and use the stochastic simulation algorithm (SSA) due to Gillespie (1976) for our stochastic simulations of Eqs. (1a), (1b). For the discrete nature of stochastic simulations, we sub-divide the domain of length *L* into *N* compartments of equal width *h*=*L*/*N*; see Fig. 4.

In the deterministic case, we choose experimentally-supported (Postma and van Haastert 2001; Postma et al. 2004) relative diffusion values *D*_*b*_=100*D*_*a*_ that effectively render *B* homogeneous. In our stochastic simulations, we approximate *B* as a homogeneous cytoplasmic pool (*D*_*b*_→∞) to reduce the computational cost, while maintaining a full computation for the heterogeneous *A* distribution. Diffusion of *A* in the membrane is treated as a series of first order reactions (spatial SSA (Erban et al. 2007)), each with propensity

$$\frac{D_{a}}{h^{2}}A_{i},\quad i=1,\ldots,N, $$

where *A*_*i*_ denotes the current number of molecules of *A* in membrane lattice point *i*. The above propensity equals zero for diffusion to the left when *i*=1, and diffusion to the right when *i*=*N*, respectively (no-flux boundary conditions). Similarly, we choose the remaining propensities according to Gillespie (1976):

$$\everymath{\displaystyle} \begin{array}{r@{\quad }l} \mbox{Background activation into membrane lattice point $i$:} & k_{0}\frac{B}{N},\\[9pt] \mbox{auto-activation of $A\,$in membrane lattice point $i$:} & \frac{\gamma A_{i}^{2}}{K_{N}^{2}+A_{i}^{2}}\frac{B}{N},\\[9pt] \mbox{background inactivation out of membrane lattice point $i$:} & \delta A_{i},\\[9pt] \mbox{pulse activation into membrane lattice point $i$:} & k_{s}\frac{B}{N}. \end{array} $$

We note that *B*, the current number of molecules of the inactive species in the well-mixed cytoplasmic pool, is rescaled with 1/*N* because each membrane lattice point only senses this fraction of the available total number of molecules of *B*. For each propensity *p*, the probability that the corresponding event occurs within the next *dt* units of time equals *p*⋅*dt*+*o*(*dt*), where *o*(⋅) denotes terms that converge to zero quicker than its argument (little o notation, $\lim_{dt\downarrow0}\frac{o(dt)}{dt}=0$).

In the above propensity expressions, most kinetic constants are equal to those used in the deterministic system, Eqs. (1a), (1b), since they are independent of the units of *a*(*x*,*t*) and *b*(*x*,*t*), or *A*_*i*_ and *B*, correspondingly. However, the Michaelis–Menten constant *K* of Eq. (2) has the same units as *a*(*x*,*t*) and needs to be rescaled for our stochastic simulations:

$$K_{N}=10^{-21}N_{A}V_{a}K, $$

where *N*_*A*_ is Avogadro’s constant, *V*_*a*_ denotes the volume associated with each membrane lattice point, $V_{a} = \frac{L}{N}W\frac{H}{2}$, and the factor 10^−21^ is required to re-scale units (*V*_*a*_ has units of μm^3^, and *K* units of μM). Homogeneous initial concentrations of *A* and *B*, *a*(*x*,0)=*a*_0_ and *b*(*x*,0)=*b*_0_, are rescaled to numbers of molecules equivalently: 
 where $V_{b}=LW\frac{H}{2}$.

Note that in our stochastic simulations we need to associate volumes with each lattice point (both for the membrane and for the cytoplasm), since it reformulates a concentration-based model, Eqs. (1a), (1b), as a molecule-based stochastic model. The natural choice for conversion between the two is through proportionality to the dimensions (volume) of the system. Even though we specify a volume for each lattice point in this conversion, the stochastic simulations are effectively one-dimensional, as are our deterministic simulations. That is, in both the deterministic and the stochastic system, we focus on radial polarisation along the diameter of a cell.

## Results

For our simulations and comparison between the stochastic and deterministic case, we use parameter values based upon Mori et al. (2008), and summarized in Table 1: *k*_0_=0.067 s^−1^, *γ*=1 s^−1^, *K*=1 μM, *δ*=1 s^−1^, and diffusion coefficients *D*_*a*_=0.1 μm^2^/s, *D*_*b*_=10 μm^2^/s (the latter in the deterministic case only). For the initial stimulus, we choose *k*_*s*_=10 s^−1^ for 50 s≤*t*≤70 s and 0 μm≤*x*≤0.4 μm, and *k*_*s*_=0 otherwise. For biologically reasonable concentrations (Marée et al. 2006), we set *b*(*x*,0)=*b*_0_=2 μM and use *a*(*x*,0)=*a*_0_ such that *f*(*a*_0_,*b*_0_)=0 and (*a*_0_,*b*_0_) is linearly stable (*a*_0_=0.2683 μM). We fix the slab height *H*=0.2 μm and length *L*=10 μm (see Fig. 1) and simulate our stochastic system for varying numbers of molecules by varying the width *W* of our cell slab. Varying the width *W* (Fig. 4) allows us to change the number of molecules without changing the initial concentrations of *A* and *B*.

**Table 1:** Summary of the parameter values used in our simulations

*D*_*a*_	0.1 μm^2^/s	diffusion of the active form *A* in the membrane
*D*_*b*_	10 μm^2^/s	diffusion of the inactive form *B* in the cytoplasm (deterministic system only)
*k*_0_	0.067 s^−1^	rate of background activation
*γ*	1 s^−1^	maximal rate of auto-activation of *A*
*δ*	1 s^−1^	rate of background inactivation
*K*	1 μM	concentration of *A* resulting in half-maximal rate of auto-activation (deterministic system)
*K*_*N*_	⋯	as constant *K* but rescaled depending on current volume of the system (*K*_*N*_∝*N*_*A*_ *HLW*⋅*K*, where *N*_*A*_ is Avogadro’s number)
*k*_*s*_	10 s^−1^	rate of activation due to transient pulse
*L*	10 μm	length of the domain
*N*	50	number of membrane lattice points

## Propagation Failure Does Not Affect the Deterministic Simulations

We first determined that the discretisation of space utilized does not cause propagation failure around the parameter values used for our analysis on stochasticity. To do so, we make use of the fact that the initial perturbation that triggers a wave does not necessarily have to be small in width. A wide (but not too wide) perturbation of sufficient amplitude can also trigger a wave. Perturbations that are wider than the final pinning position, however, trigger waves with a negative velocity, i.e. waves that retract until they come to halt at the pinning position. If indeed propagation failure plays a role, both the extending and the retracting wave are expected to halt before their velocities would have become zero in the continuous case. Thus, propagation failure would cause both waves to a halt at distinct positions.

**Fig. 5:**
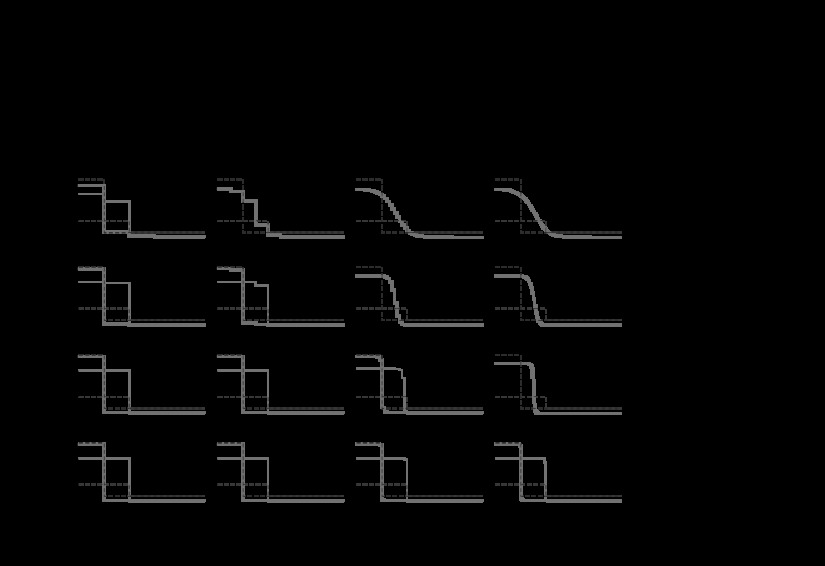
*Propagation failure in the deterministic system as a function of grid coarseness and diffusion coefficient of the active form*. For each column a different number of compartments, *N*, has been used, and for each row a different diffusion coefficient, *D*_*a*_. The values of both are indicated in the figure, with diffusion coefficient *D*_*a*_ in units μm^2^/s. *Dashed lines* denote initial conditions, *solid lines* are equilibrium profiles (at *t*=20,000 s), *thick red lines* denote narrow, high initial waves, *thin blue lines* denote broad, shallow initial waves. Discrepancies between the *red* and *blue* equilibrium profiles for slow diffusion coefficients and coarse grids can be attributed to propagation failure. At our default setting of *D*_*a*_=0.1 μm^2^/s and *N*=50, propagation failure is not observed (Color figure online)

We therefore devised the following numerical experiment: We use a total amount of *T*=22.68, our default value, from Mori et al. (2008), and within the wave-pinning regime, in which a *Δ*-perturbation is required to trigger a wave that gives rise to sustained polarity; see the black arrow in Fig. 3. We then start simulations, for varying values of the active form diffusion coefficient *D*_*a*_ and number of lattice sites *N*, with two different wave-shaped initial conditions (see Fig. 5): one narrower and higher, one broader and lower, both indicated by dashed lines in the figure, where the former lies to the left and the latter to the right of the equilibrium wave-pinning position.

Figure 5 shows the equilibrium wave profiles for the narrowly initiated waves (thick red lines) and broadly initiated waves (thin blue lines). Indeed, sufficiently large box sizes (low *N*) and sufficiently low diffusion coefficients show a discrepancy in the final position of the pinned wave between the retracting and the extending waves, illustrating how too slow diffusion combined with too coarse sub-compartmentalization leads to propagation failure. For the default values used in this study (*N*=50; *D*_*a*_=10^−1^ μm^2^/s), however, no propagation failure can be observed. A 10-fold coarser grid or 100-fold slower diffusion would be needed for propagation failure to occur.

## Equilibrium Behaviour of the Stochastic System

**Fig. 6:**
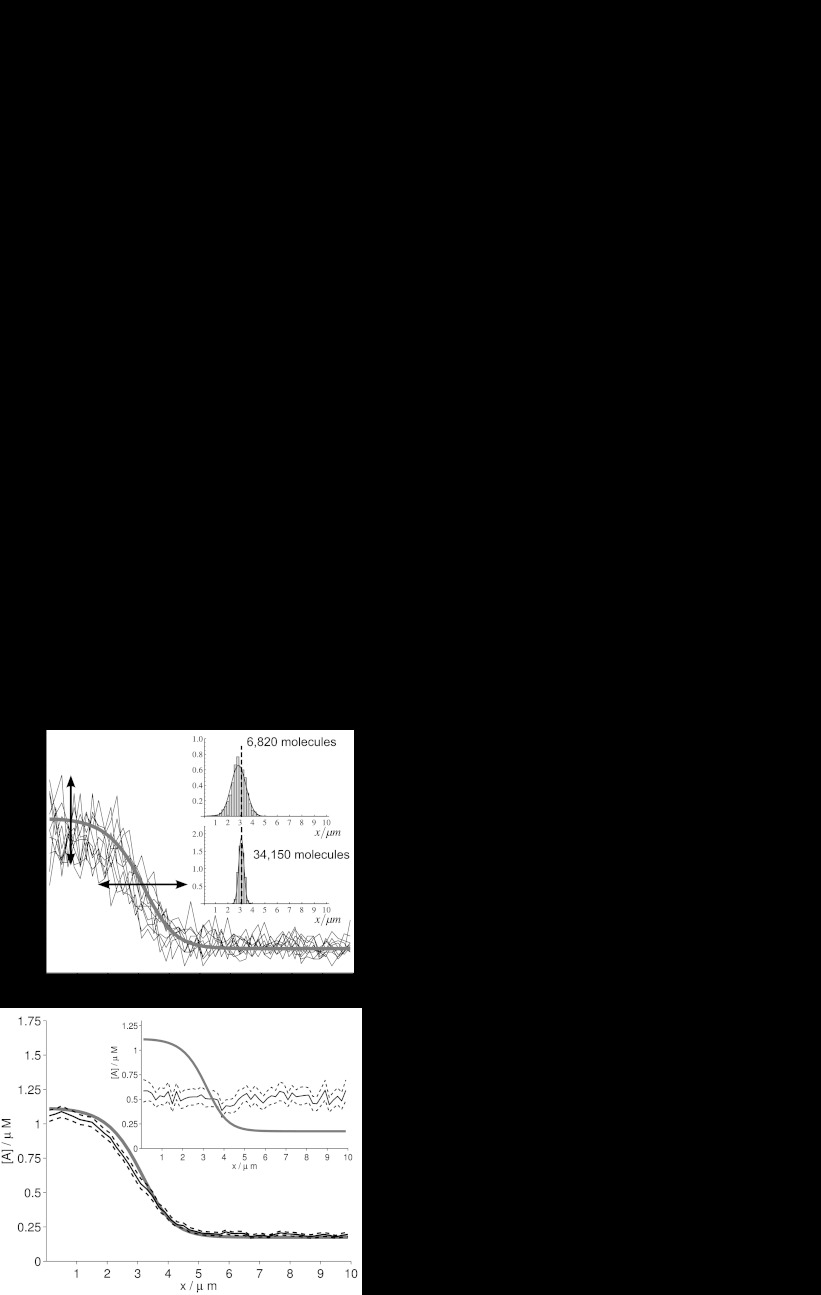
*Stochasticity in wave-pinning*. (*Top*) *Black solid lines*: ten individual runs of the stochastic model using the SSA at *t*=200 s. Vertical and horizontal noise of the *jagged lines* are indicated by the corresponding *arrows*. *Gray line*: behaviour of the deterministic system. (Parameter values *k*_0_=0.067 s^−1^, *γ*=1 s^−1^, *K*=1 μM, *δ*=1 s^−1^, *D*_*a*_=0.1 μm^2^/s, *D*_*b*_=10 μm^2^/s; diffusion of *B* in deterministic model only). Total amount *T*=22.68. *W*=2.5 μm, number of molecules equals 6,820. (*Insets*) Histograms of observed pinning positions when number of molecules is 6,820 and 34,150, respectively. Concentration profiles of 100 simulation runs between 150 and 200 s were fitted to symmetric Richards model and inflection point used as pinning position. (*Bottom*) *Black solid line*: observed mean of the stochastic system over 100 runs, shown together with a *grey line* representing the deterministic system. *Black dashed lines* enclose the 95 % c.i. of the sample mean. (*Bottom inset*) *Lines* as in *bottom image*, and parameters as in *top image* except for width, *W*=0.25 μm, resulting in a reduction of the number of molecules to 702. The *top image* shows that individual SSA runs closely reproduce the deterministic behaviour for appropriate parameters. The corresponding mean behaviour in the *bottom image* confirms this. The *bottom inset* depicts loss of wave-pinning from the mean behaviour when too few molecules are present in the system

Individual SSA runs (Fig. 6, top) resemble the deterministic behaviour for sufficiently large numbers of molecules (≈6800), and the averaged concentration profile clearly shows wave-pinning after a stimulus is applied (Fig. 6, bottom). With fewer molecules (≈700), individual SSA runs become more erratic and the averaged profile is homogeneous across the domain, resulting in loss of wave-pinning (Fig. 6, bottom, inset). As we increase the numbers of molecules by increasing *W*, we observe convergence of the stochastic system to the deterministic one, indicated by a decreasing observed variance and smoothing of the black solid curve. To study the distribution of pinning positions, we fit, well after wave-pinning, the concentration profile of the active species to the symmetric Richards model (Richards 1959), which very well captures sigmoidal profiles and contains a parameter, *c*, that defines the position of the inflection point, which we consider to be the pinning position of the wave:

$$a_{\text{fit}}(x)=M \biggl(1-\frac{1}{1+\exp(-(x-c)/h)} \biggr)+m, $$

where *M* and *m* are the concentrations at the high and low plateau, respectively, *h* determines the slope at the inflection point, and *c* is the position of the inflection point. The insets in Fig. 6, top, show the distribution in pinning position for the indicated number of molecules, based upon concentration profiles from 100 simulation runs collected between 150 s and 200 s and recorded every 0.1 s (i.e. based upon 50, 100 concentration profiles). We fit it to a normal distribution, indicated by the black line. At high molecule numbers, the distribution closely follows a Gaussian distribution, with the mean corresponding to the predicted value from the PDE analysis (indicated by the dashed line). At low molecule numbers, however, we find that the distribution becomes leptokurtic, illustrating that the wave is more confined to its pinning position than is to be expected from the level of variation observed. Also, at small molecule numbers, the pinning position becomes slightly biased to smaller *L*_0_^∗^ (i.e. to the left), due to the fact that stochasticity also creates an effectively lower level of auto-activation; see the discussion below. At a high molecule number, fluctuations in the pinning position become small and closely spaced around the position within the deterministic model (indicated by the dashed line). At lower molecule numbers, the profile broadens.

To quantify convergence of the stochastic case, *A*_*i*_(*t*), to the deterministic case, *a*_*i*_(*t*), at time *t* and lattice point *i*, we use the normalized Euclidean distance

5 $$ d(t)=\sqrt{\sum_{i=1}^{N}{ \bigl[A_{i}(t)-a_{i}(t)\bigr]^{2}}}\bigg/\sqrt {\sum_{i=1}^{N}{a_{i}(t)^{2}}} . $$

We compute this distance for *J*=100 SSA runs, *d*(*t*)^(*j*)^ (*j*=1,…,*J*), and report mean observed distances $\overline{d(t)}=(1/J)\sum_{j=1}^{J}{d(t)^{(j)}}$.

**Fig. 7:**
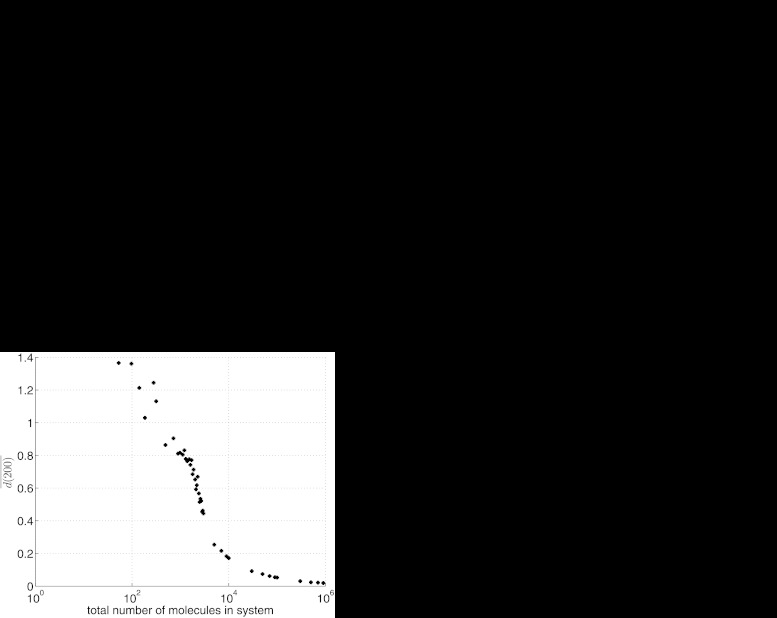
*Convergence of stochastic to deterministic system*. Mean Euclidean distance over *J*=100 SSA runs, $\overline{d(200)}$ at time *t*=200 s, after the stimulus. Simulation parameters as in Fig. 6, except for *W*. (*W* is varied to obtain different total copy numbers.) This plot shows convergence of the stochastic system to macroscopic predictions as we increase the number of molecules

The observed mean Euclidean distance in Fig. 7 shows the expected convergence of the stochastic case to the deterministic case for increasing copy number. In Fig. 7, disappearance of the wave profile becomes apparent by the increasing value of $\overline{{d(200)}}$ for fewer molecules. These results show the stochastic limit to the deterministic behaviour, confirming the qualitative intuition of a stochastic lower limit to the wave pinning mechanism. What is not clear a priori, however, is the dynamics by which wave-pinning is lost at low molecule numbers and how this is affected by other parameter changes.

## Stochastic Simulations Are Not Affected by Propagation Failure

**Fig. 8:**
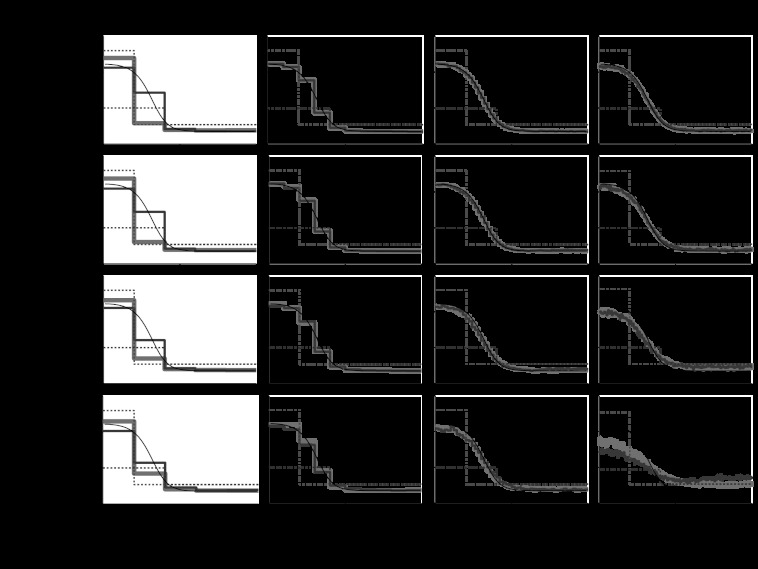
*Propagation failure within stochastic simulations as a function of grid coarseness and molecule number*. *Dashed profiles* indicate initial conditions, correspondingly *coloured solid lines* depict mean equilibrium profiles at *t*=200 s, averaged over 100 runs. Confidence intervals are omitted for reasons of clarity, but standard errors of mean are similar to Fig. 6. *D*_*a*_=0.1 μm^2^/s; the number of compartments and molecules are indicated in the figure. Propagation failure does not affect 50 lattice point simulations. At small molecule numbers, however, larger numbers of lattice points present deviations that are unlike propagation failure (Color figure online)

Before we discuss in detail the loss of wave-pinning at low molecule numbers, we revisit the phenomenon of propagation failure. We repeated the procedure described above to determine potential propagation failure within the stochastic description, but now for varying molecule numbers rather than diffusion rates (Fig. 8). Indeed, as before, when the grid becomes rougher, secondary effects of propagation failure manifest themselves and affect the pinning positions through this independent mechanism. Again however, as we showed before in Fig. 5, those effects are not noticeable in the simulation regimes we focused on, and only to a very small extent under extreme conditions of a very coarse grid.

We then studied how stochasticity influenced this relationship. We found that stochasticity reduces rather than increases the parameter regime for which propagation failure can be observed (compare the column corresponding to *N*=5 in Fig. 8 with the upper left graph in Fig. 5). This can be understood by realizing that stochasticity can help overcome the threshold to propagate the wave. This means that for coarse grids and low diffusion rates the stochasticity at low molecule numbers can even increase the precision of the pinned wave. Besides deviations at low box numbers, we also observe deviations from the stationary solution of the deterministic model (as indicated in each frame with a black line) for a combination of low molecule and high box numbers (see lower right graph in Fig. 8). This is an artefact attributed to an effective change in the auto-activation function when the number of molecules in a box becomes very small, which will be discussed further below.

## Comparison of Deterministic and Stochastic Predictions

We test our predictions from the LPA system, Eqs. (4a), (4b), by classifying individual SSA runs (using the same parameter values as in Fig. 6) as either homogeneous in *A* (i.e. uniform in *A* well after the stimulus, e.g. at *t*=200 s) or inhomogeneous in *A* (i.e. where a local pulse invaded the global concentration profile of *A*, creating at least one high plateau or peak in *A*).

**Fig. 9:**
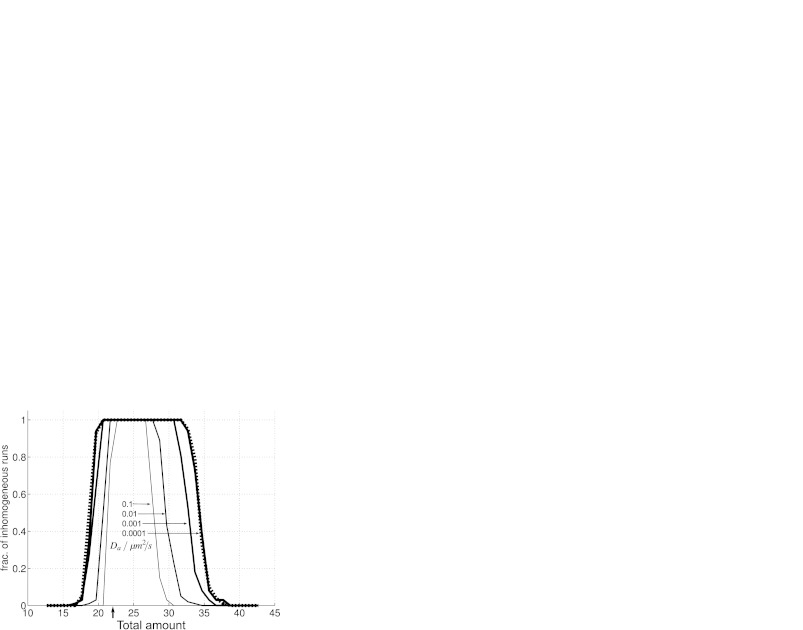
*Dependence of wave-pinning stability on diffusion and total amount*. Fraction of SSA runs (out of 100 runs) observed at *t*=200 s to be stably inhomogeneous (wave-pinned, or with multiple peaks in *A*). System parameters as in Fig. 6 with varying *D*_*a*_. Each inverted-cup-shaped curve corresponds to a different *D*_*a*_ value, decreasing from *D*_*a*_=0.1 μm^2^/s to *D*_*a*_=10^−5^ μm^2^/s from the innermost to the outermost cup, using order of magnitude changes in the parameter value (as indicated by the *arrows*). *Dashed line*: *D*_*a*_=0 μm^2^/s. This plot shows for what parameter values (changing total amounts *T* and diffusion constant *D*_*a*_) the stochastic system shows stabilization of perturbations in the uniform concentration profile of *A* (high fraction of inhomogeneous runs). Stabilization of perturbations is difficult for high diffusivity of *A* (*D*_*a*_=0.1 μm^2^/s innermost, narrowest cup) since spikes in the concentration profile of *A* are more readily smoothed out before they grow to sufficiently high levels. For decreasing values of *D*_*a*_, our observations match progressively better with the predictions made using the LPA in the main text. That is because the LPA assumes zero diffusivity for *A* and, therefore, ignores any effects due to the diffusion of the active form

Comparing the predictions of the deterministic version of the model with the SSA simulations we find interesting differences. With parameter values as in Fig. 6, specifically with *D*_*a*_=0.1 μm^2^/s (central inverted cup in Fig. 9), we observe a somewhat more stringent condition in the stochastic wave-pinning regime, 22≤*T*≤27, than predicted by Fig. 3 (arrow indicates default value *T*=22.68). This discrepancy may be explained by the relatively fast diffusion of *A*, compared with the limiting rates used in the theoretical treatment, which destabilizes local perturbations in *A* (through high curvature in the concentration profile of *A*), making it harder for perturbations to stabilize and invade the remaining profile of *A*. Indeed, as we decrease *D*_*a*_ by orders of magnitude (Fig. 9, broadening inverted cups), increasingly wider ranges of *T* allow for stabilization of perturbations in *A* and their invasion of the global homogeneous *A* level. Our observations for decreasing *D*_*a*_, and certainly for *D*_*a*_=0 μm^2^/s (Fig. 9, dashed line), indicate increasing agreement between our stochastic simulations and the predicted wave-pinning criteria.

## Loss of Wave-Pinning in Small Number Regimes

Loss of wave-pinning for fewer molecules may either result from increased randomness along the concentration axis (vertical noise, Fig. 6), or greater random fluctuations in the pinning position of a travelling wave (horizontal noise, Fig. 6): With a low copy number, individual runs may show wave-pinning but the steepest point of the concentration profile (pinning position) may fluctuate along the horizontal axis due to inherent stochasticity.

To distinguish the effects caused by these distinct types of noise, we quantify the horizontal noise at lattice point *i*, using a sample of *J*=100 SSA runs, with a function similar to auto-correlation, here denoted “spatial auto-correlation”:

6 $$ RS_{k}(i)=\frac{\mathbb{E} [ (A_{i}(t)-\overline{\mathbb{E}[A(t)]} ) (A_{i}(t+k)-\overline{\mathbb{E}[A(t+k)]} ) ]}{\overline{\operatorname{Var} [A_{i} ]}}, $$

where *k* is the time lag, $\overline{\mathbb{E}[A(t)]}$ is the spatial mean of *A* at time *t*, and $\overline{\operatorname{Var}[A_{i}]}$ is a spatial variance. (We use bars to denote statistics related to spatial auto-correlation.) Statistic $\overline{\mathbb{E}[A(t)]}$ measures the average concentration of *A* at time *t* across the domain, and the spatial variance, $\overline{\operatorname{Var}[A_{i}]}$, gauges the spread of the concentration of *A* at lattice point *i*, *A*_*i*_(*t*), about the spatial mean. Observing the concentration of *A* in *M* equidistant time points (*A*_*i*_(*m*), *m*=1,…,*M*), we estimate these statistics as follows: 
7a
7b Our estimator of spatial auto-correlation then becomes:

8 $$ \widehat{RS}_{k}(i)=\frac{\frac{1}{M-k}\sum_{m=1}^{M-k}{[A_{i}(m)-\hat{\overline{\mu}}(m)] [A_{i}(m+k)-\hat{\overline{\mu}}(m+k)]}}{\hat{\overline{\sigma}_{i}}^{2}}. $$

This function centres observations *A*_*i*_(*m*) about the spatial mean and gauges the correlation between observations *k* time steps apart. We also normalize our estimator ($\widehat{RS}_{k}(i)\in(-1,1)$) by dividing by the observed variance about the spatial mean. A high-value $\widehat{RS}_{k}(i)$ denotes a lattice point *i* where *A*_*i*_ is stably far away from the spatial mean, while a low value suggests that *A*_*i*_ repeatedly comes near the spatial mean.

We report estimates of auto-correlation (spatial, and temporal further on) as sample means $\widehat{\overline{RS_{k}}}(i)= (1/J)\sum_{j=1}^{J}{\widehat{RS_{k}}(i)^{(j)}}$ of the corresponding estimates for *J* SSA runs, $\widehat{RS_{k}}(i)^{(j)}$. We further compute these estimates in time periods when we expect our system to be stable, long after the application of a stimulus and wave-pinning (stimulus applied between 50 s and 70 s and observations between 500 s and 1500 s used for computations).

**Fig. 10:**
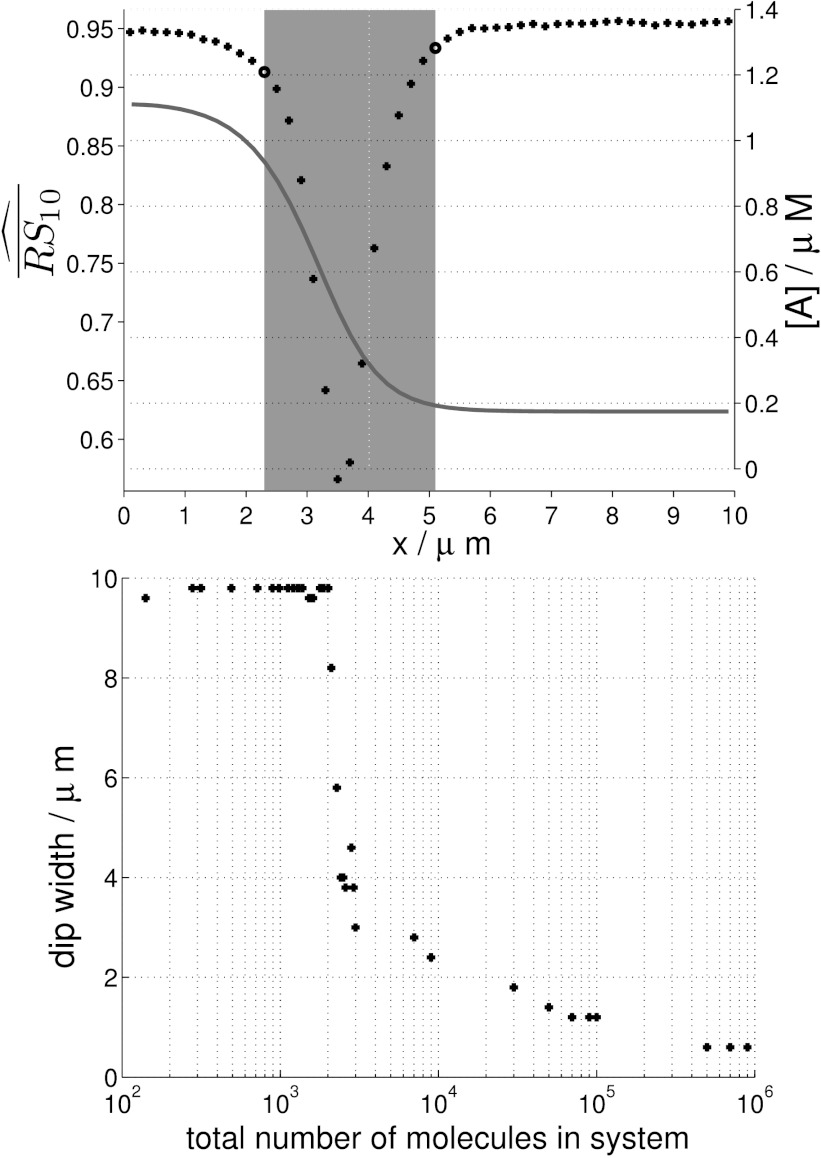
*Noisy transition zone*. (*Top*) Averaged spatial auto-correlation estimate, Eq. (8), lag *k*=1 s, with deterministic solution as in Fig. 6 for reference. *Bold circles*: delimiters of auto-correlation dip, highlighted by *shaded area*. This figure shows correspondence of a dip in spatial auto-correlation with the transition zone of the pinned wave. (*Bottom*) As in *top figure*, mean auto-correlation dip width (width of *grey rectangle* in *top figure*) over 100 SSA runs, as a function of total number of molecules. When the dip width approaches the length of the domain of the cell (10 μm), the pinning position stops conferring information that can be used by the cell. We note a sudden and drastic increase in width at approximately 2,000 molecules. Simulation parameters as in Fig. 6, except for *W* which is varied

In a regime where the mean behaviour presents wave-pinning, we observe a dip in spatial auto-correlation in a section of the domain that includes the pinning position (Fig. 10, top). The width of this dip (shaded area in Fig. 10, top) increases with decreasing numbers of molecules in the system (Fig. 10, bottom), and spans the entire domain (10 μm) when wave-pinning is lost.

For quantifying vertical noise, we use an auto-correlation function denoted “temporal auto-correlation” for clarity:

9 $$ RT_{k}(i)=\frac{\mathbb{E} [ (A_{i}(t)-\mathbb{E}[A_{i}] ) (A_{i}(t+k)-\mathbb{E}[A_{i}] ) ]}{\operatorname{Var} [A_{i} ]^{2}}, $$

where $\mathbb{E}[A_{i}]$ is the expected value and $\operatorname{Var}[A_{i}]^{2}$ is the variance of concentration *A*_*i*_. We use the common sample mean and sample variance as estimators of these statistics: 
10a
10b Using these, our estimator of temporal auto-correlation becomes

11 $$ \widehat{RT_{k}}(i)=\frac{\frac{1}{M-k}\sum_{m=1}^{M-k}[A_{i}(m) -\hat{\mu}_{i}][A_{i}(m+k)-\hat{\mu}_{i}]}{\hat{\sigma}_{i}^{2}}. $$

**Fig. 11:**
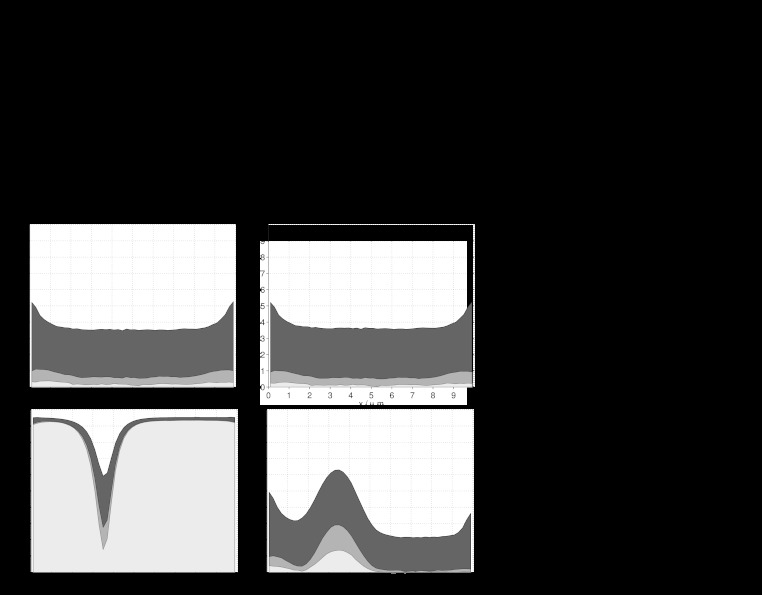
*Fluctuations in space and time*. Spatial/temporal auto-correlation sample mean (over *J*=100 SSA runs) with lag *k*=1 s (*dark grey*), *k*=10 s (*grey*), and *k*=20 s (*light grey*). (*Top row*) 702 molecules (*W*=0.25 μm). (*Top left*) Spatial auto-correlation. (*Top right*) Temporal auto-correlation. (*Bottom row*) 6,820 molecules (*W*=2.5 μm). (*Bottom left*) Spatial auto-correlation. (*Bottom right*) Temporal auto-correlation. The plots in the *top row* show that for sufficiently few molecules both spatial and temporal autocorrelation are almost uniform and show no pattern for the equilibrium state (pinning position). In the *bottom row* and for a sufficiently high number of molecules, spatial auto-correlation reveals a clear pattern in the equilibrium state with the dip in autocorrelation robust across different lags *k*. In the same copy-number regime, temporal auto-correlation shows a pattern for small lags *k* which, however, vanishes for increasing values of *k*. Simulation parameters as in Fig. 6, except for *W*

Temporal auto-correlation measures the randomness of the time evolution of *A*, which is governed by a continuous-time Markov process. (If we know the distribution of *A*(*t*) at present, the future distribution of *A*(*s*), *s*>*t*, only depends on *A*(*t*) and is independent of observations of *A* before *t*.) Given the Markovian character of *A*(*t*), we expect the temporal auto-correlation to be greatly dependent on the lag *k*: a small lag (*k*=1 s) results in relatively high temporal auto-correlation (black area, Fig. 11 top right and bottom right), while a bigger *k*=20 s yields small temporal auto-correlation (light grey area, Fig. 11 top right and bottom right). We also observe that the Markovian character of *A*(*t*) is comparable for small and large numbers of molecules since the magnitude of temporal auto-correlation does not change significantly when altering the number of molecules (Fig. 11 right panels). This means that in both small and large copy regimes, temporal auto-correlation decreases comparably fast.

While the vertical noise does not seem to decrease when increasing the number of molecules, wave-pinning still shows up as a marked peak in temporal auto-correlation in the transition zone of the wave (Fig. 11, bottom right). Since overall vertical noise is relatively constant, this peak seems to be caused by the wave fluctuating about its pinning position: concentration *A*_*i*_(*t*) in the transition zone fluctuates between high and low values (high and low plateau of wave) causing *A*_*i*_(*t*) to be far away from its average repeatedly (high temporal auto-correlation).

We find spatial auto-correlation to behave markedly different from temporal autocorrelation for increasing numbers of molecules (Fig. 11, left panels). While spatial auto-correlation is comparable in magnitude to temporal auto-correlation in a small copy number regime (Fig. 11 top panels), we observe much greater spatial auto-correlation than temporal auto-correlation for various lag *k* values in a large copy number regime (Fig. 11 right panels). The stably high spatial auto-correlation, even for large lags *k*, far away from the pinning position suggests that the high and low plateau of the wave are persistent in wave-pinning regimes (Fig. 11 bottom right). The random fluctuation of the pinning position is highlighted by the decreasing spatial auto-correlation for increasing lags *k* (dark to light grey area in Fig. 11 bottom left) in this part of the domain.

**Fig. 12:**
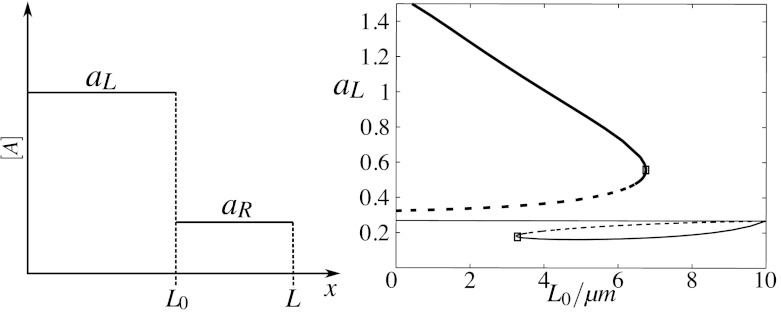
*Polarised cell state analysis.* Schematic of the simplified deterministic wave-shaped state, consisting of a high plateau *a*_*L*_ of length *L*_0_ on the *left*, and a low plateau *a*_*R*_ of length *L*−*L*_0_ on the *right*. (*Right*) Bifurcation plot of steady-state concentrations of *a*_*L*_ as a function of *L*_0_, with parameters as in Fig. 6 and with total amount *T*=22.68. Two saddle-node bifurcations occur at critical values  and . The corresponding bifurcation plot of *a*_*R*_ is symmetric to this one of *a*_*L*_. In our stochastic simulations, for equivalent parameter values and number of molecules = 6,820, we did not observe pinning positions greater than 6 μm (*top inset*, Fig. 6), as is predicted by this analysis

To elucidate the source of the horizontal noise and the impact it has on the sustainability of a polarised cell, we make use of another spatially simplified representation of the deterministic system. Without loss of generality, once a wave has been formed, the concentration profile of *a* can be split into a plateau of length *L*_0_ and approximate concentration *a*_*L*_ on the left, and a plateau of length *L*−*L*_0_ and approximate level *a*_*R*_ on the right (Fig. 12 left) where

$$a(0\leq x<L_{0},0)=a_{L}(0)=a_{1}, \qquad a(L_{0} \leq x\leq L,0)=a_{R}(0)=a_{2}. $$

Here, *a*_1_>*a*_2_ are two roots of *f*(*a*,*b*_*wp*_)=0, where *b*_*wp*_ is the uniform concentration of *b* for this wave-shaped profile, while it is assumed that *D*_*b*_=∞ and *D*_*a*_=0. Then the total amount *T* of protein in the domain can be approximated as *T*_*wp*_=*Lb*_*wp*_+*L*_0_ *a*_1_+(*L*−*L*_0_)*a*_2_. In the limit *D*_*a*_→0 (no direct communication between plateaus), *a*_*L*_(*t*) and *a*_*R*_(*t*) evolve independently. Eliminating *b*(*t*) using mass conservation leads to:

12 $$ \everymath{\displaystyle}\begin{array}{rcl} \frac{da_{L}}{dt} & = & f(a_{L},b), \qquad\frac{da_{R}}{dt}=f(a_{R},b), \\[12pt] b(t) & = & (T_{wp}/L)-a_{L}(t) (L_{0}/L)-a_{R}(t) (L-L_{0})/L, \end{array} $$

which also implies that the position of the wavefront is

13 $$ L_{0}=\frac{T_{wp}-L[b(t)+a_{R}(t)]}{a_{L}(t)-a_{R}(t)}. $$

System (12) is a second deterministic reduction that leads to a way of comparing the stochastic and deterministic model versions. In the deterministic case, we expect that the wave-pinned configuration, Fig. 12, left panel, is stable over time when *L*_0_=*L*_0_^∗^ (pinning position). As Eq. (13) indicates, noise in the concentration variables will propagate to the width of the high plateau of the pinned wave. We expect that this propagation of noise is qualitatively the same in the full spatial system and conjecture that noise in the pinning position (horizontal noise) is the result of noise in the concentration levels (vertical noise).

Given that the total amount is fixed at *T*_*wp*_, *a*_*L*_ and *a*_*R*_ adjust to varying widths of *L*_0_: increasing *L*_0_ will typically decrease *a*_*L*_ and increase *a*_*R*_ accordingly, and vice versa. For system (12), we plot the steady-state concentration of *a*_*L*_ as a function of *L*_0_ (Fig. 12, right panel) and observe two critical values for *L*_0_, $L_{0}^{(1)} < L_{0}^{(2)}$, at which saddle-node bifurcations occur. The bifurcation plot in Fig. 12 shows that if we initialize our simplified system in a wave-pinned configuration (high plateau on left, low plateau on right, as in Fig. 12, left panel, with $L_{0}={L_{0}}^{*} < L_{0}^{(2)}$), sufficiently large horizontal noise may drive the effective plateau width, *L*_0_, away from *L*_0_^∗^ and past the critical point ($L_{0} > L_{0}^{(2)}$) which would then cause the wave to collapse. Due to hysteresis in the bifurcation plot, *L*_0_ may fluctuate back to the left of $L_{0}^{(2)}$ after collapse of the wave without triggering a restoration of the wave.

We expect that the saddle-node bifurcation at $L_{0}^{(2)}$ and hysteresis explain the sharp increase in dip width (Fig. 10, bottom) observed in the full spatial system: decreasing the number of molecules in the system increases vertical noise which propagates into horizontal noise. As horizontal noise increases, the likelihood that the pinning position randomly overshoots the critical value increases and we are more likely to observe wave collapse (dip width approaching 10 μm, Fig. 10, bottom). This prediction is further supported by our observation that under conditions equivalent to those of Fig. 12, and with 6,820 molecules, we do not observe any pinning positions greater than 6 μm (Fig. 6, top inset).

**Fig. 13:**
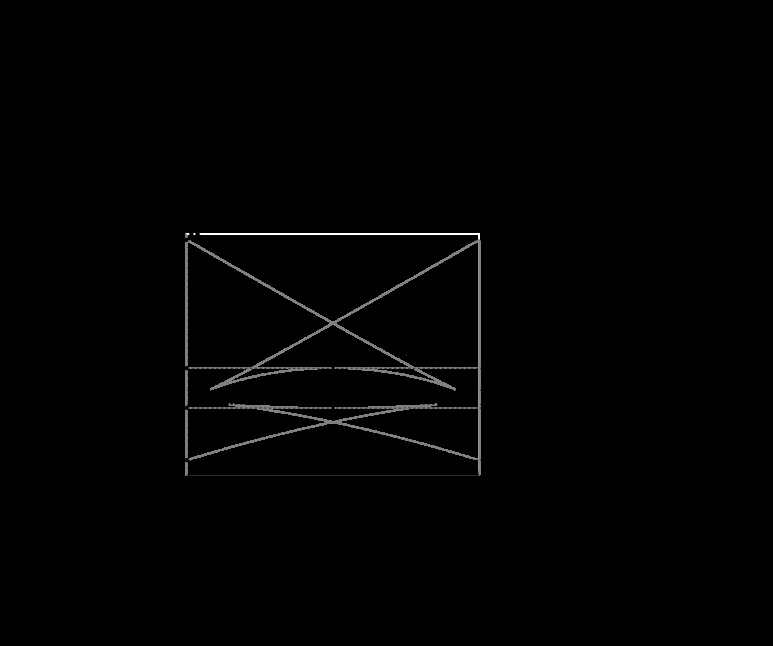
*Two-parameter bifurcation plot of system* (12). This figure shows the connection between the LPA (Fig. 3) and the loss-of-wave-pinning analysis (Fig. 12). It also presents the requirements on both height and breadth of an initial *Δ*-perturbation to trigger cell polarity. (*Top right*) Two-parameter bifurcation plot, with total amount *T* along *y*-axis and wave position *L*_0_ along *x*-axis. *Grey lines* starting at FB: fold bifurcation lines; grey lines starting at TB: transcritical bifurcation lines; CP: cusp bifurcation; FT: Collision of fold and transcritical bifurcation. (*Surrounding sub-figures*) When varying *T*, seven distinct zones of qualitatively different behaviour are found, labelled I–VII. To understand the behaviour in each zone, bifurcation diagrams are plotted of the equilibrium value *a*_*L*_ (Eq. (12)) as a function of *L*_0_ for a fixed value of *T* within that zone

Figure 12 is closely linked to Fig. 3. While the latter shows the impact of a *Δ*-perturbation of infinitesimally small width, corresponding to an *L*_0_=0 μm, as a function of *T*, the former shows the impact of perturbations of varying width, for a fixed value of *T*. The link between the analysis on the loss of wave-pinning with system (12) and the analysis on polarity initiation (LPA, system (4a), (4b)) is very direct. By allowing *L*_0_↓0, system 12 becomes equivalent to system (4a), (4b): *a*_*L*_ in Eq. (12) describes the active level on a vanishingly small domain and, therefore, ceases to affect the inactive species *b* (i.e. *a*_*L*_—active left—becomes the local perturbation *a*_*L*_—active local—in Eqs. (4a), (4b)), while *a*_*R*_ starts occupying the entire domain of length *L* and, therefore, only the presence of *a*_*R*_ affects *b* (i.e. *a*_*R*_—active right—becomes the global active form *a*_*G*_—active global—in Eqs. (4a), (4b)). Figure 13 brings both pieces of information together, showing a two-parameter bifurcation plot, with *L*_0_ along the *x*-axis, and *T* along the *y*-axis. The right panel of Fig. 12 corresponds to a horizontal cross-section at *T*=22.68 (Fig. 13, zone VI), while the bifurcation diagram of Fig. 3 corresponds to a vertical cross-section through this bifurcation diagram at *L*_0_=0, along which line we have indicated the two fold bifurcations (FB) and two transcritical bifurcations (TB) that can be seen in Fig. 3, indeed reproduced at exactly the same parameter values.

Increasing *L*_0_ away from zero, the critical total amounts of both fold bifurcations in Fig. 3 change while the critical values of the transcritical bifurcations are unaffected (solid grey lines in Fig. 13). It implies that at levels of *T* that are less favourable for sustained polarity, narrow *Δ*-perturbations are still able to trigger a wave, while broad ones can not do that any more. Moreover, it illustrates that when the well-mixed equilibrium is unstable against spatially inhomogeneous bifurcations (zones III–V), the width of the perturbation becomes irrelevant. We also observe four cusp bifurcation points (CP), describing where two fold bifurcation lines merge. These points imply the possible coexistence of waves with different amplitudes. Finally, there are two bifurcations where a fold bifurcation and a transcritical bifurcation collide (FT). They are linked to the symmetry of the two-parameter bifurcation plot about *L*_0_=5 μm, which is due to the lack of inherent bias for either a left-oriented or a right-oriented polarisation in system (12): when *L*_0_<5 μm, the pinned wave has its high plateau on the left, while for *L*_0_>5 μm the high plateau is on the right. Due to this symmetry, we for example observe a bifurcation plot equivalent to Fig. 3 when plotting *a*_*R*_ and setting *L*_0_=10 μm (data not shown), confirming that initiation of a wave from the left is equivalent to initiation from the right. Together, Fig. 13 reveals that there are seven qualitatively different zones of levels of *T*, each presenting diverse requirements on wave initiation and maintenance. It allows us to predict the potential to trigger (or sustain, when *L*_0_=*L*_0_^∗^) a wave through a perturbation of any possible width and height, as well as the expected height that such a wave will reach while it travels, stalls, or stochastically fluctuates. Specifically, it sets the boundaries for horizontal fluctuations to trigger a collapse of the polarised cell state.

## Validity of Stochastic Model at Large Compartment Numbers

**Fig. 14:**
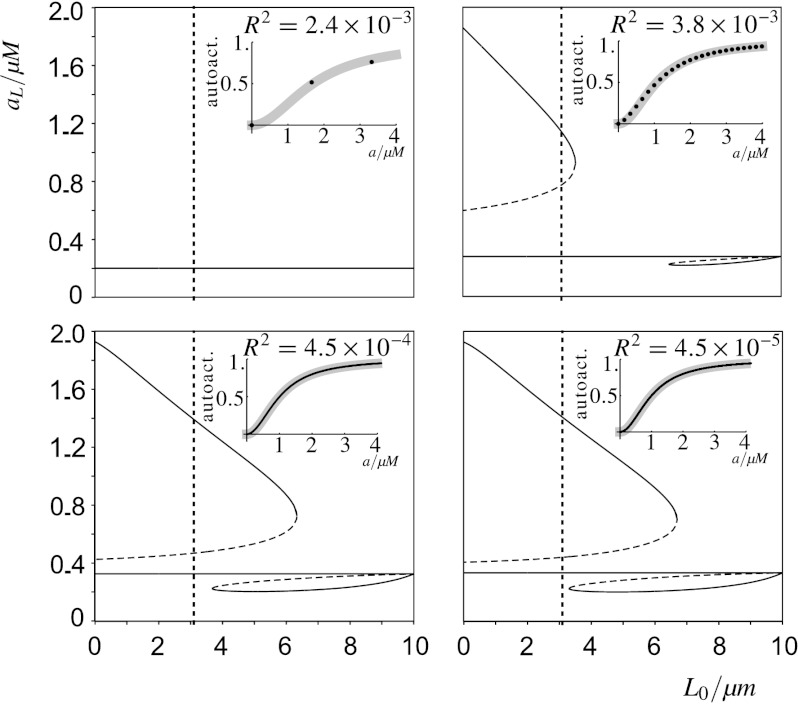
*Predicted effect of stochasticity on the auto-activation term and its consequences for wave-pinning*. Bifurcation diagrams to analyse the polarised cell state, as in Fig. 12, for four different box volumes *V*_*a*_ (*top left*: 0.001 μm^3^; *top right*: 0.01 μm^3^; *bottom left*: 0.1 μm^3^; *bottom right*: 1.0 μm^3^). The different bifurcation diagrams are made by varying the parameter values *γ* and *K* of the Hill-type auto-activation *γa* ^2^/(*K* ^2^+*a* ^2^). The effective parameter values *γ* and *K* for small box sizes are determined as follows: For the mean number of molecules of active *A* being equal to 0,1,2,… molecules per box (indicated by the *black dots* in the *insets*), a Poisson distribution for that number of molecules is assumed in order to calculate an expected rate of auto-activation. These observed values are then fitted to the auto-activation term itself using a least-squares fit. The fitted auto-activation functions are shown as *grey lines* in the *insets*, together with the residual sums of squares, *R* ^2^. We observe that while the fitted *γ* hardly differs from the originally used value (*γ*≈1), the fitted *K* increases as box volume decreases. The bifurcation diagrams show that this change in effective auto-activation lowers the higher plateau and narrows the range of permissible *L*_0_ values. Note that the difference between the *lower panels* is virtually undetectable. Indeed, both are almost equivalent to the bifurcation diagram for the deterministic system, which was shown in Fig. 12. It illustrates that a volume of *V*_*a*_=0.1 μm^3^ (*bottom left*) forms the upper bound for observing this stochastic effect. This corresponds to 137 molecules per box, given that the total concentration is 2.268 μM. The lower bound for wave-pinning to occur is reached when the effective *K* becomes 1.07, at a box size of *V*_*a*_=0.009 μm^3^, corresponding to 14 molecules per box

Sub-dividing the domain into *N* compartments is a computational method which should not influence the biological insights derived here. Above we discussed that the coarseness of the lattice could introduce artefacts due to propagation failure, but that the simulations in this study are sufficiently fine-grained. Figure 8, however, also presented deviations from the expected wave profile when *N* was large. When testing the behaviour of the stochastic model as *N*→∞, we realized that this is due to the fact that the effective auto-activation function starts to change as the number of molecules in a box becomes very small. Given that at high *N* only small variations between the mean values of neighbouring boxes are expected (as the flux between boxes goes to infinity), we tested if the change in auto-activation could be due to the expected stochastic variations within each box, assuming a Poisson distribution for the number of active molecules within each box. We therefore calculated the predicted mean activation rate as a function of the concentration *a* for different box sizes, by taking the kernel of the Poisson distribution with the auto-activation function itself. The insets of Fig. 14 show the resulting auto-activation rates for different box sizes, to which subsequently the auto-activation function $\frac{\gamma a^{2}}{K^{2}+a^{2}}$ was fitted, with both *K* and *γ* as the fitting parameters. The figure shows that when the number of active molecules becomes small the functional response effectively shifts to the right (i.e. the fitted value of *K* becomes larger), while the plateau hardly changes (i.e. *γ* remains more or less 1.0). This phenomenon is due to the plateau in the sigmoidal activation function: while the high spectrum of the Poisson distribution cannot further increase the activation rate, as it is capped, the low spectrum decreases it. Figure 14 repeats the analysis of Fig. 12 for the estimated effective values of *K*. It shows that consequently at lower molecule numbers the wave becomes lower and is lost more easily due to horizontal fluctuations until, at very low molecule numbers per box, the wave cannot be sustained any longer. Also, the lower critical value of *L*_0_, $L_{0}^{(2)}$, shifts to lower values (i.e. to the left). This shift to lower *L*_0_ and drop in height of the wave closely corresponds to the observed changes in the stochastic simulations as can be seen in Fig. 8, suggesting that the side-effects observed when increasing *N* are due to this modification of the auto-activation function only. This reduction in the number of molecules per box when increasing *N* can be overcome in our modelling set-up by increasing *W*. Thus, *N* can be made arbitrarily large, as long as, through modifications of *W*, the number of molecules per box is maintained above around 14 (below which value the effective *K* becomes too large to warrant sustainable wave-pinning).

## Discussion

In this paper, we compared deterministic and stochastic aspects of a model for cell polarisation of the wave-pinning class (Mori et al. 2008). This work was motivated by recent interest in the influence of stochastic noise in biological systems. In considering stochastic noise, we account for possible effects due to low-copy numbers of signalling proteins, as has been done for instance by Isaacson et al. (2011).

Recent studies indicate that noise can have either constructive or detrimental effects in biological systems. For example, noting beneficial effects, Paulsson et al. (2000) observed that stochastic focusing increased sensitivity of cascades, Rao et al. (2002) found that noise-induced population heterogeneity improves fitness, Howard and Rutenberg (2003) argued that biologically relevant oscillations in a two-component dynamical system are more robust in the stochastic case than the deterministic one, and Gamba et al. (2005) showed that stochasticity could play a role in chemotactic responses to shallow gradients. On the other hand, detrimental effects were noted by, for example, McAdams and Arkin (1997) who showed that gene expression in a noisy regime resulted in bursts, rather than constant levels of gene expression. For our stochastic model of cell polarisation, we observe that at critically low molecule numbers stochastic noise has an impact on the behaviour of the system in a detrimental manner, eventually destroying polarisation.

As shown in Fig. 6, we verify correctness of our stochastic implementation by comparing our stochastic simulation results in large copy number regimes with the deterministic system (approaching the macroscopic limit). The compartmentalized spatial Gillespie model used here, however, does present deviations when the lattice number *N* becomes very small or very large. Alternatively, an off-lattice Brownian dynamics model could have been developed (Andrews and Bray 2004; van Zon 2005), to independently confirm the results based upon space discretisation presented here, given that both approaches present their own limitations (Erban and Chapman 2007, 2009). However, to re-formulate the auto-activation in terms of mass-action kinetics only, which is required to use off-lattice alternatives, would require a replacement of the current parsimonious non-linear term by, for example specific enzyme kinetics. Such a description would involve the introduction of new variables and new assumptions as well as relatively ad hoc choices regarding their reactions and behaviour, obfuscating the comparison between the PDE model and the stochastic model.

Here, we studied wave-pinning in a one-dimensional slab of cell material representing radial positional information available to a single cell. Our results suggest that for small copy numbers, radial information within a cell regarding its front and back that is available through the wave-pinning process decreases in quality because of fluctuations of the wave around its pinning position. If we extrapolate our results to a spherical 15 μm-diameter cell and assuming micro-molar small G-protein concentration (corresponding to 10^6^ molecules), stable polarisation should typically be observed. However, in vivo effective reaction compartment size is often restricted due to macro-molecular crowding and resulting volume exclusion (Schnell and Turner 2004; Grima 2010): the volume encompassed by a cell is generally occupied by a range of macro-molecules which do not participate in any of the relevant chemical reactions. As these macro-molecules span the cell in a mesh-like fashion, individual effective reaction compartments may emerge which have a potentially small volume. Our results indicate that sufficiently small effective reaction compartments (those that hold 10^3^ molecules, bottom Fig. 10), will produce inaccurate positional information (fluctuation of the pinning position). Hence, a cell subject to great amounts of macro-molecular crowding may integrate inaccurate positional information from its individual effective reaction compartments and, therefore, lose a global sense of directionality.

Instead of a gradual loss of wave-pinning, we observe a threshold number of molecules (between 2,000 and 3,000 molecules) below which the wave is suddenly lost. Analysis of a limiting deterministic case shows that sudden loss of wave-pinning is due to saddle-node bifurcations with hysteresis. We conjecture that this also explains sudden loss of polarisation in our spatial stochastic system.

Altschuler et al. (2008) studied a related positive feedback model of Cdc42 with a homogeneous cytoplasmic pool of the inactive form. Their model includes self-recruitment of the active form, but the conditions for polarisation and wave-pinning as defined through the LPA method described in this paper are not fulfilled, hence no stable polarisation can be observed. Nevertheless, by defining polarisation as a transient situation where 10 % of the domain holds more than 50 % of the molecules, they were able to show that within their model decreasing the numbers of molecules, which increases the random fluctuations, could trigger polarisation. This is opposite to what has been shown in our study, in which increasing fluctuations shuts off the polarity. For a fixed positive feedback strength, they found that 1,000 molecules yields the maximum probability (fraction of simulation runs) for polarisation. For 3,000 molecules or more, they observed that fewer than 50 % of the runs would polarise. In contrast, our model predicts that polarisation fails below 2,000 molecules (Fig. 10, bottom panel). Moreover, in our model, the behaviour of the stochastic system more closely resembles that of the deterministic system when the molecule number increases (bottom panel of Fig. 6), i.e. unlike Altschuler et al. (2008) with more molecules the polarity becomes increasingly more robust.

Khain et al. (2011) recently discussed stochastic travelling waves in a spatial one-dimensional model of spruce budworm populations, in which the high plateau of the wave corresponded to parts of the environment with a great number of budworms (outbreak state) and the low plateau denoted few budworms (refuge state). Note that in their model wave-pinning does not occur. Nevertheless, they compared a deterministic version (thermodynamic limit) of their model with a stochastic version of it and observed differences in wave propagation velocity. They explained that these differences are caused by random jumps, possible within the stochastic system, from the high plateau to the low plateau and vice versa, similar to our explanation for the fluctuations observed in the pinning position. In their case, however, the stochasticity affects the velocity of the travelling wave, while in our study it affects the pinning position.

In future efforts, it would be interesting to study the stochastic model for cell polarisation in higher dimensions, where effects of geometry are non-trivial (e.g. see Strychalski et al. 2010), as well as in off-lattice Brownian dynamics models.

Finally, studies such as this one can be extended to tackle the intriguing question of how cells communicate polarisation within a wider tissue context, which is a subject of ongoing work. We envision that stochasticity within the coupling of cell polarities between cells could play an important role.

### Numerical Methods

Plots in Figs. 5 and 8, histograms and fits in inset of Fig. 6, and least-squares fitting and plots of insets of Fig. 14 were done with Mathematica (Version 8.0, Wolfram Research, Inc., Champaign, IL, USA). Stochastic and deterministic simulations and plots of Figs. 1, 6, 7, 9, 10, and 11 were done with MATLAB (2010a, The MathWorks, Natick, MA, USA). The two-parameter plot of Fig. 13 was done with MATLAB and matcont (Dhooge et al. 2003). Simulations and bifurcation plots of top two rows in Fig. 3, and bifurcation plots of Fig. 12, insets of Fig. 13, and Fig. 14 were done with XPP-AUTO (G.B. Ermentrout, University of Pittsburgh).

## References

[CR1] Altschuler S. J., Angenent S. B., Wang Y., Wu L. F. (2008). On the spontaneous emergence of cell polarity. Nature.

[CR2] Andrews S. S., Bray D. (2004). Stochastic simulation of chemical reactions with spatial resolution and single molecule detail. Phys. Biol..

[CR3] Britton N., Sleeman B., Jarvis R. (1985). Travelling wave front solutions of a differential-difference equation arising in the modelling of myelinated nerve axon. Ordinary and partial differential equations.

[CR4] Charest P. G., Firtel R. A. (2007). Big roles for small GTPases in the control of directed cell movement. Biochem. J..

[CR5] Dhooge A., Govaerts W., Kuznetsov Yu. A. (2003). MATCONT: A MATLAB package for numerical bifurcation analysis of ODEs. ACM Trans. Math. Softw..

[CR6] Erban R., Chapman S. J. (2007). Reactive boundary conditions for stochastic simulations of reaction-diffusion processes. Phys. Biol..

[CR7] Erban R., Chapman S. J. (2009). Stochastic modelling of reaction-diffusion processes: algorithms for bimolecular reactions. Phys. Biol..

[CR8] Erban, R., Chapman, J., & Maini, P. (2007). A practical guide to stochastic simulations of reaction-diffusion processes. arXiv:0704.1908v2. 10.1088/1478-3975/4/1/003 17406082

[CR9] Fáth G. (1998). Propagation failure of traveling waves in a discrete bistable medium. Physica D.

[CR10] Gamba A., De Candia A., Di Talia S., Coniglio A., Bussolino F., Serini G. (2005). Diffusion-limited phase separation in eukaryotic chemotaxis. Proc. Natl. Acad. Sci. USA.

[CR11] Gillespie D. (1976). A general method for numerically simulating the stochastic time evolution of coupled chemical reactions. J. Comput. Phys..

[CR12] Grima R. (2010). Intrinsic biochemical noise in crowded intracellular conditions. J. Chem. Phys..

[CR13] Howard M., Rutenberg A. (2003). Pattern formation inside bacteria: fluctuations due to the low copy number of proteins. Phys. Rev. Lett..

[CR14] Isaacson S. A., McQueen D. M., Peskin C. S. (2011). The influence of volume exclusion by chromatin on the time required to find specific DNA binding sites by diffusion. Proc. Natl. Acad. Sci. USA.

[CR15] Jilkine A., Marée A. F. M., Edelstein-Keshet L. (2007). Mathematical model for spatial segregation of the Rho-family GTPases based on inhibitory crosstalk. Bull. Math. Biol..

[CR16] Keener J. P. (1987). Propagation and its failure in coupled systems of discrete excitable cells. SIAM J. Appl. Math..

[CR17] Khain E., Lin Y. T., Sander L. M. (2011). Fluctuations and stability in front propagation. Europhys. Lett..

[CR18] Li Z., Hannigan M., Mo Z., Liu B., Lu W., Wu Y., Smrcka A. V., Wu G., Li L., Liu M. (2003). Directional sensing requires G*βγ*-mediated PAK1 and PIX*α*-dependent activation of Cdc42. Cell.

[CR19] Marée A. F. M., Jilkine A., Dawes A., Grieneisen V. A., Edelstein-Keshet L. (2006). Polarization and movement of keratocytes: a multiscale modelling approach. Bull. Math. Biol..

[CR20] McAdams H. H., Arkin A. (1997). Stochastic mechanisms in gene expression. Proc. Natl. Acad. Sci. USA.

[CR21] McQuarrie D. A. (1967). Stochastic approach to chemical kinetics. J. Appl. Probab..

[CR22] Mori Y., Jilkine A., Edelstein-Keshet L. (2008). Wave-pinning and cell polarity from a bistable reaction-diffusion system. Biophys. J..

[CR23] Mori Y., Jilkine A., Edelstein-Keshet L. (2011). Asymptotic and bifurcation analysis of wave-pinning in a reaction-diffusion model for cell polarization. SIAM J. Appl. Math..

[CR24] Paulsson J., Berg O. G., Ehrenberg M. (2000). Stochastic focusing: fluctuation-enhanced sensitivity of intracellular regulation. Proc. Natl. Acad. Sci. USA.

[CR25] Postma M., van Haastert P. J. M. (2001). A diffusion-translocation model for gradient sensing by chemotactic cells. Biophys. J..

[CR26] Postma M., Bosgraaf L., Loovers H. M., Van Haastert P. J. M. (2004). Chemotaxis: signalling modules join hands at front and tail. EMBO Rep..

[CR27] Raftopoulou M., Hall A. (2004). Cell migration: Rho GTPases lead the way. Dev. Biol..

[CR28] Rao C. V., Wolf D. M., Arkin A. P. (2002). Control, exploitation and tolerance of intracellular noise. Nature.

[CR29] Richards F. J. (1959). A flexible growth function for empirical use. J. Exp. Bot..

[CR30] Ridley A. J. (2006). Rho GTPases and actin dynamics in membrane protrusions and vesicle trafficking. Trends Cell Biol..

[CR31] Ridley A. J., Schwartz M. A., Burridge K., Firtel R. A., Ginsberg M. H., Borisy G., Parsons J. T., Horwitz A. R. (2003). Cell migration: integrating signals from front to back. Science.

[CR32] Schnell S., Turner T. E. (2004). Reaction kinetics in intracellular environments with macromolecular crowding: simulations and rate laws. Prog. Biophys. Mol. Biol..

[CR33] Strychalski W., Adalsteinsson D., Elston T. C. (2010). Simulating biochemical signaling networks in complex moving geometries. SIAM J. Sci. Comput..

[CR34] van Zon J. S., ten Wolde P. R. (2005). Green’s-function reaction dynamics: a particle-based approach for simulating biochemical networks in time and space. J. Chem. Phys..

